# Validation of clinical frailty scale in Chinese translation

**DOI:** 10.1186/s12877-022-03287-x

**Published:** 2022-07-20

**Authors:** Yi-Chun Chou, Hsiao-Hui Tsou, Ding-Cheng Derrick Chan, Chiung-Jung Wen, Feng-Ping Lu, Kun-Pei Lin, Meng-Chen Wu, Yung-Ming Chen, Jen-Hau Chen

**Affiliations:** 1 Department of Geriatrics and Gerontology, National Taiwan University Hospital, No.7, Chung Shan South Road, Taipei, 100 Taiwan; 2 Institute of Population Health Sciences, National Health Research Institutes, 35 Keyan Road, Zhunan, Miaoli County 350 Taiwan; 3 Department of Internal Medicine, College of Medicine, National Taiwan University, No.1, Jen Ai Road Section 1, Taipei, 100 Taiwan; 4 Department of Family Medicine, College of Medicine, National Taiwan University, No.1, Jen Ai Road Section 1, Taipei, 100 Taiwan; 5 Department of Neurology, National Taiwan University Hospital, No.7, Chung Shan South Road, Taipei, 100 Taiwan; 6 Superintendent Office, National Taiwan University Hospital Bei-Hu Branch, No. 87, Neijiang Street, Taipei, 108 Taiwan

**Keywords:** Clinical frailty scale, Frailty, Validation studies, Elderly

## Abstract

**Background:**

Identification of frailty is crucial to guide patient care for the elderly. The Clinical Frailty Scale (CFS) is a reliable, synthesis and clinical judgment-based tool. However, a validated Chinese version of CFS (CFS-C) is lacking. The aim of this study is to describe the translation process of CFS into traditional Chinese and to evaluate its reliability and validity in a geriatric study population in Taiwan.

**Methods:**

This cross-sectional study recruited 221 geriatric outpatients aged 65 years or older at a medical center in Taipei, Taiwan. The Chinese version of CFS was produced following Brislin’s translation model. Weighted kappa for agreement and Kendall’s tau for correlation were used to assess inter-rater reliability (a subgroup of 52 outpatients) between geriatricians and one research assistant, and validity tests (221 outpatients) by comparing CFS-C with Fried frailty phenotype and Frailty Index based on Comprehensive Geriatric Assessment (FI-CGA). Correlation between CFS-C and other geriatric conditions were also assessed.

**Results:**

The inter-rater reliability revealed moderate agreement (weighted kappa = 0.60) and strong correlation (Kendall’s tau = 0.67). For criterion validity, CFS-C categorisation showed fair agreement (weighted kappa = 0.37) and significant correlation (Kendall’s tau = 0.46) with Fried frailty phenotype, and higher agreement (weighted kappa = 0.51) and correlation (Kendall’s tau = 0.63) with FI-CGA categorisation. CFS-C was significantly correlated with various geriatric assessments, including functional disability, physical performance, hand grip, comorbidity, cognition, depression, and nutrition status. No significant correlation was found between CFS-C and appendicular muscle mass.

**Conclusions:**

The CFS-C demonstrated acceptable validity and reliability in Chinese older adults in Taiwan. Development of CFS-C enhanced consistency and accuracy of frailty assessment, both in research and clinical practice.

**Supplementary Information:**

The online version contains supplementary material available at 10.1186/s12877-022-03287-x.

## Background

Frailty has become an emerging concern as the population ages worldwide with its prevalence varied from 4 to 59% according to different measures [1]. It is a state of decreased reserve capacity which leads to vulnerability to various stressors [2] and associates with increased risk for falls, fractures, disability, institutionalization, hospitalization and death [3–8]. As frailty indicates more about the aging process than chronological age alone, its assessment can help to identify older adults at risk and corresponding interventions [9]. Because of its dynamic and potential reversible nature, early identification of frailty is crucial to guide patient care for elderly with different degrees of frailty [10, 11].

Currently, there is no single standard definition of frailty. There are several operational instruments of frailty mainly derived from two approaches: frailty phenotype by Fried et al. and the Frailty Index (FI) of accumulation deficits by Rockwood et al. [11, 12]. The former defines frailty by using five standardized, physiologically based signs and symptoms, and the latter defines frailty by counting age-related deficits (at least 30), including not only signs and symptoms but also diseases and disabilities [2, 11, 13]. These two concepts, representing different aspects of frailty, are considered as complementary rather than substitutable [14]. However, measurement of grip strength and gait speed in frailty phenotype or collecting data of FI is sometimes time-consuming in clinical settings [12].

By contrast, the Clinical Frailty Scale (CFS) by Rockwood et al. has been adapted into a relatively quick, reliable and clinical judgment-based tool. A 7-point version was originally developed for the Canadian Study of Health and Ageing (CSHA) and was highly correlated with FI [15]. It was further updated as a 9-point version (CFS version 1.2) [16]. CFS was associated with mortality, comorbidity, cognition, falls, and function [17]. In this pandemic era, CFS was also associated with mortality in coronavirus disease 2019 (COVID-19) with dose–response relationship and was recommended as a tool for individualized assessment of frailty to manage COVID-19 in older adults [18, 19].

Validated CFS studies in different languages have been published, such as French, Danish, Greek, and Korean [20–24]. CFS usage is also growing in the Asia–Pacific region [12]. Since Taiwan is one of the fastest-ageing countries in the world, domestic policies have evolved to prioritize health and social care for older adults. CFS is the recommended tool for frailty assessment in integrated outpatient services in Taiwan and now serves as one of the inclusion criteria for the post-acute care program [25, 26]. As practice guidelines recommend identifying frailty using validated frailty measurement tools, validation of a Chinese version of CFS is warranted [12].

We previously validated a simplified telephone version in Chinese of the CSHA CFS for rapid screening of frail elders in the community [27]. However, it was adapted from the original 7-point CFS, and was never formally translated. Some differences, including considerations of cognition, pattern of disabilities, and life expectancy, existed between the 7-point and 9-point CFS [16]. Therefore, for more accurate assessment of different levels of frailty, the aim of this study is to describe the translation process of the 9-point CFS into Chinese (traditional Chinese) and to evaluate its reliability and criterion concurrent validity by comparing against two main instruments of frailty: Fried frailty phenotype and FI.

## Methods

### Study population and design

This was a cross-sectional validation study using data from a prospective cohort study recruiting geriatric outpatients at the National Taiwan University Hospital (NTUH) in Taipei, Taiwan. Data was collected between June and December 2019. The inclusion criteria were age ≥ 65 years and having at least one of the following geriatric syndromes: fall or functional decline in recent one year, polypharmacy ≥ 5, urinary incontinence, history of osteoporosis or weight loss (≥ 5% in one month or 10% in 6 months). Our study excluded patients with severe dementia, severe hearing or visual impairment, severe functional impairment or contact precautions for multidrug resistant organisms in order to avoid communication or cooperation barriers. The study was approved by the Research Ethics Committee at NTUH. Written informed consent of the study participants was obtained before enrollment.

### Sample size calculation

For inter-rater reliability, we assumed the minimum acceptable kappa was 0.2 and anticipated a substantial agreement (weighted Kappa = 0.61–0.80) between CFS-C of physicians and one research assistant. At least 48 participants were required for assuring a power of 80% and a significant level of 0.05 to detect a statistically significant kappa coefficient [28, 29]. For criterion validity, we assumed the minimum acceptable Kendall’s tau correlation was 0.2 and expected a high correlation (Kendall’s tau > 0.3) between CFS-C and Fried frailty phenotype. Thus, at least 211 participants were required for assuring a power of 80% and a significance level of 0.05 to detect a statistically significant Kendall’s tau coefficient [30]. Allowing 5–10% attrition rate for missing data, our study enrolled 226 geriatric outpatients. After exclusion of 5 participants who had no CFS-C assessment (*n* = 4) or no BabyBot vital data (*n* = 1), a total of 221 subjects were included for criterion validity and 52 of them were included for reliability analysis [see Additional file 1].

### Data collection

A wide range of demographic and health data was collected on BabyBot vital data recording system (Netown Corporation, Taiwan) and comprehensive geriatric assessment (CGA). BabyBot included a 68-item self-reported questionnaire, bioelectrical impedance analysis (Tanita BC-418), and tests of hand grip, timed-up and go (TUG), and 6-m walk. CGA, comprised of Mini-Mental State Examination (MMSE) [31], Geriatric Depression Scale-15 (GDS-15) [32], Mini-Nutritional Assessment (MNA) [33], Barthel Index (BI) [34], and Instrumental Activities of Daily Living (IADL) [35], was evaluated by a trained research assistant. To measure comorbidity, six geriatricians scored the Cumulative Illness Rating Scale for Geriatrics (CIRS-G) [36].

### Translation of the clinical frailty scale into Chinese

**Fig. 1:**
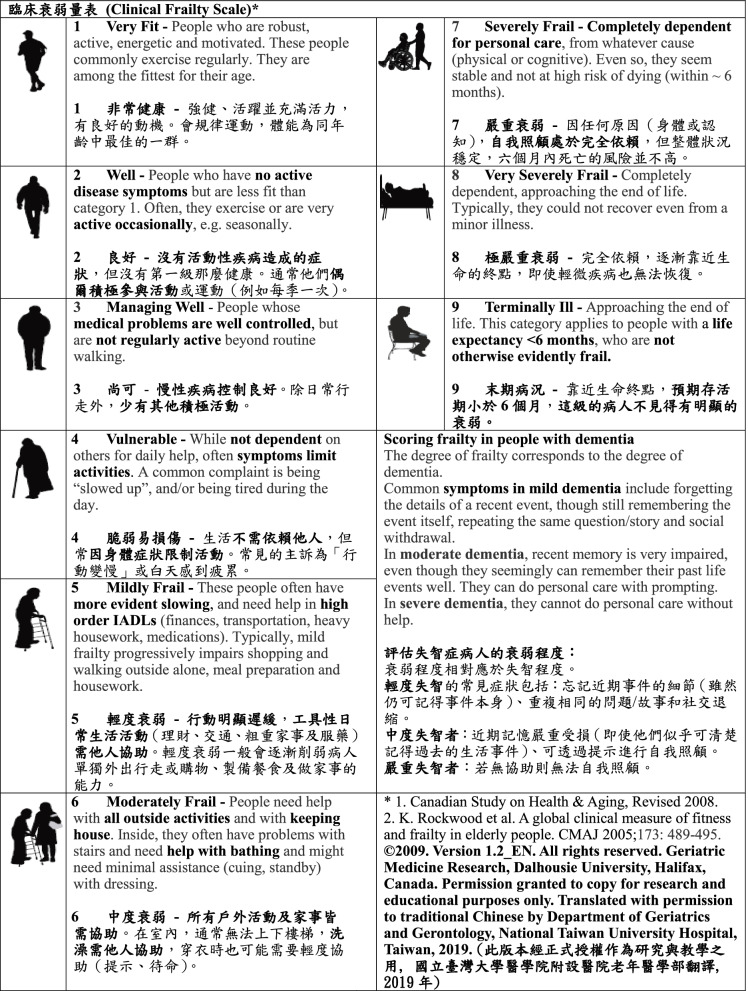
Chinese version of Clinical Frailty Scale (CFS-C)

With Dr. Rockwood’s permission, we undertook the translation process following Brislin’s translation model [37, 38]. To start, the English version CFS (referred to as the source CFS) was translated into traditional Chinese by one of the authors of this study, as well as by a bilingual translator working independently. The two translated CFS documents were evaluated and compared with the source CFS by a panel of experts (seven geriatricians and one nurse practitioner) to reach consensus. Afterwards, back translation was independently conducted by two bilingual primary care physicians who had never seen the source CFS. Lastly, three bilingual experts and a panel of geriatric experts were involved in group discussion to compare the two back translations with the source CFS. Minor discrepancies were resolved, and the expert reviewers agreed on the production of the final Chinese version of CFS (CFS-C, Fig. 1).

### Assessment of frailty

#### The Chinese version of Clinical Frailty Scale (CFS-C)

The CFS-C was scored by the same trained research assistant after completing BabyBot and CGA. For the reliability group of 52 participants, CFS-C was scored independently and simultaneously by their geriatricians after reviewing the results of BabyBot and CGA. The results of CFS-C were blinded to each other. For criterion concurrent validity, CFS-C was categorised as robust (CFS-C = 1–2), prefrail (CFS-C = 3–4) and frail (CFS-C = 5–9) [17].

#### The Fried frailty phenotype

Fried frailty phenotype was assessed by five criteria: exhaustion, weight loss, low activity, weakness, and slowness [2]. We assessed presence of exhaustion, weight loss or low activity by reporting of a “yes” answer to the following items in the self-reported questionnaire: “Feeling tired or fatigue in recent one month”, “weight loss of more than 3 kg or 5% in the previous year” and “low physical activity”, respectively. Weakness was determined by having low grip strength below established cut-off (< 28 kg in men, < 18 kg in women) [39]. Slowness was defined as gait speed < 1 m/s based on the 6-m walk or the participant was not able to walk [39]. From a 5-point scale, participants scored 0 were defined as non-frail, scored 1 or 2 as prefrail, and scored ≥ 3 as frail.

#### Frailty Index based on a Comprehensive Geriatric Assessment (FI-CGA)

FI-CGA gathered information on ten standard domains from CGA and BabyBot, including cognition, emotion, communication, mobility, balance, bladder function, bowel function, nutrition, activities of daily living and social resources [40–42]. For each domain, “0” indicated no problem, “0.5” indicated a minor problem, and “1” indicated a major problem. Scores were summed up into an impairment index, ranging from 0 to 10. For co-morbidity index, CIRS-G was standardized to a range from 0 to 4, representing equivalence of 4 deficits. To construct FI-CGA, the sum of the impairment and co-morbidity index were further divided by 14 into a range from 0 to 1. The detailed scoring criteria were presented in Table 1. According to previous reported cutoffs, participants were categorised as robust (FI-CGA ≤ 0.08), prefrail (0.08 < FI-CGA < 0.25) and frail (FI-CGA ≥ 0.25) [43].

**Table 1:** Frailty Index based on a Comprehensive Geriatric Assessment (FI-CGA)

Domains	Scoring methods	Data source
1. Cognition	0—Normal MMSE	CGA
	0.5—Abnormal MMSE^a^ and normal IADL and BI	
	1—Abnormal MMSE and (IADL or BI)	
2. Emotion	0—GDS < 5	CGA
	0.5—5 ≤ GDS < 10	
	1—GDS ≥ 10	
3. Communication	0—No deficit in communication, hearing, vision	Questionnaire from Babybot
	0.5—1 deficit in either communication, hearing, vision	
	1—≥ 2 deficits in either communication, hearing, vision	
4. Mobility	0—TUG < 10	TUG test from Babybot
	0.5—10 ≤ TUG ≤ 19	
	1—TUG > 19 or unable to walk	
5. Balance	0—No self-reported poor balance and no fall in previous year	Questionnaire from Babybot
	0.5—Report of either fall in previous year or poor balance	
	1—Report fall and poor balance	
6. Bladder	0—Bladder control in BI = 10	CGA
	0.5—Bladder control in BI = 5	
	1—Bladder control in BI = 0	
7. Bowel	0—Bowel control in BI = 10	CGA
	0.5—Bowel control in BI = 5	
	1—Bowel control in BI = 0	
8. Nutrition	0—MNA = 12–14	CGA
	0.5—MNA = 8–11	
	1—MNA = 0–7	
9. ADL	0—IADL = 8 and BI = 100	CGA
	0.5—IADL < 8 and BI = 100	
	1—BI < 100	
10. Social resources	0—Not living alone and someone could help if needed	Questionnaire from Babybot
	0.5—(Living alone but someone could help if needed) or (not living alone but no one could help if needed)	
	1—Living alone and no one could help if needed	
Impairment Index = sum of deficits (numbers of deficits = 0–10)		
Comorbidity Index = CIRS-G standardized to 0–4 (numbers of deficits = 0–4)		
FI-CGA = (Impairment Index + Comorbidity Index)/14		

*Abbreviations*: *MMSE* Mini-Mental State Examination, *CGA* Comprehensive geriatric assessment, *IADL* Instrumental Activities of Daily Living, *BI* Barthel Index, *GDS* Geriatric Depression Scale, *TUG* Timed-up and go, *MNA* Mini-Nutritional Assessment, *ADL* Activities of Daily Living, *CIRS-G* Cumulative Illness Rating Scale for Geriatrics, *FI-CGA* Frailty Index based on a Comprehensive Geriatric Assessment

^a^ Abnormal MMSE was defined as MMSE ≤ 23 if years of education > 2 years or MMSE ≤ 13 if years of education ≤ 2 years

### Statistical analysis

Descriptive analysis was presented as numbers (%) for categorical data, and mean ± standard deviation for continuous variables. Weighted kappa for agreement and Kendall’s tau for correlation were used to assess inter-rater reliability and validity tests. Inter-rater reliability was assessed between physicians and the research assistant. For criterion concurrent validity, CFS-C was compared with both Fried frailty phenotype and FI-CGA. Kendall’s tau was used to assess correlation between CFS-C and other geriatric assessments, including BI, IADL, MNA, MMSE, GDS, CIRS-G, 6-m gait speed, TUG, hand grip and appendicular skeletal muscle mass (ASM). Data was analyzed by using SAS version 9.4 (SAS Institute Inc., Cary, NC). A two-sided *p* < 0.05 was set as statistically significance.

## Results

### Characteristics of the study population

Among 221 participants analyzed in the validation study, the mean age was 80.5 ± 7.1 years with a range from 65 to 97 years. Three-fifths (59%) of them were female, 53% had at least ≥ 9 years of education and half were classified as overweight or obese (BMI ≥ 24 kg/m^2^). For frailty assessment, the classification of CFS-C ranged from 1% (category 1) to 31% (category 4). None of the participants were classified as category 8 or 9. When using Fried frailty phenotype and FI-CGA, 53% and 56% of the participants were classified as frail, respectively. Other characteristics of the study population were presented in Table 2.

**Table 2:** Baseline characteristics of the study participants (*n* = 221)

	n (%) Mean, SD
Age (years old)	80.5, 7.1
Female	130 (58.8)
Education (years)	8.9, 5.0
BMI (kg/m^2^)	24.5, 4.0
**Frailty assessment**	
CFS-C	
1	2 (0.9)
2	28 (12.7)
3	43 (19.5)
4	69 (31.2)
5	29 (13.1)
6	41 (18.5)
7	9 (4.1)
Fried frailty phenotype	
Robust	15 (7.0)
Prefrail	86 (40.4)
Frail	112 (52.6)
FI-CGA	0.3, 0.2
FI-CGA ≤ 0.08	17 (7.9)
0.08 < FI-CGA < 0.25	78 (36.5)
FI-CGA ≥ 0.25	119 (55.6)
**Elements of geriatric assessments**	
BI	88.4, 20.9
IADL	5.5, 2.8
MNA	11.5, 2.3
0–7	16 (7.2)
8–11	73 (33.0)
12–14	132 (59.7)
MMSE	22.2, 5.8
Abnormal^a^	100 (45.5)
Normal^a^	120 (54.5)
GDS	4.8, 4.0
< 5	120 (54.8)
5–9	61 (27.9)
≥ 10	38 (17.4)
CIRS-G	11.9, 5.3
**Elements of BabyBot**	
ASM^b^ (kg/m^2^)	7.6, 1.4
ASM^b^ (Male)	8.7, 1.2
ASM^b^ (Female)	6.8, 0.8
6-m gait speed (m/s)	1.0, 0.4
TUG test (seconds)	18.4, 11.3
Hand grip (kg)	16.8, 6.9
Hand grip (Male)	21.1, 7.1
Hand grip (Female)	13.7, 4.7

*Abbreviation*: *SD* Standard deviation, *BMI* Body Mass Index, *CFS-C* Chinese version of Clinical Frailty Scale, *FI-CGA* Frailty Index based on a Comprehensive Geriatric Assessment, *BI* Barthel Index, *IADL* Instrumental Activities of Daily Living, *MNA* Mini-Nutritional Assessment, *MMSE* Mini-Mental State Examination, *GDS* Geriatric Depression Scale, *CIRS-G* Cumulative Illness Rating Scale for Geriatrics, *ASM* Appendicular skeletal muscle mass, *TUG* Timed-up and go

^a^ Abnormal MMSE was defined as MMSE ≤ 23 if years of education > 2 years or MMSE ≤ 13 if years of education ≤ 2 years

^b^ ASM was adjusted using height squared

### Inter-rater reliability

Of 52 participants in the reliability group, the inter-rater reliability revealed moderate agreement (weighted kappa = 0.60) and strong correlation (Kendall’s tau = 0.67). All *p* values were < 0.0001 (Table 3).

**Table 3:** Reliability and validation tests of CFS-C

Tests	N	Weighted kappa	*p*-value	Kendall’s tau	*p*-value
Inter-rater reliability					
Physicians vs. research assistant	52	0.60	< .0001	0.67	< .0001
Criterion concurrent validity					
CFS-C categorisation^a^ vs. Fried frailty phenotype	213	0.37	< .0001	0.46	< .0001
CFS-C vs. FI-CGA	214	-	-	0.64	< .0001
CFS-C categorisation^a^ vs. FI-CGA categorisation^b^	214	0.51	< .0001	0.63	< .0001

*Abbreviations*: *CFS-C* Chinese version of Clinical Frailty Scale, *FI-CGA* Frailty Index based on a Comprehensive Geriatric Assessment

^a^ Robust: CFS-C 1–2, prefrail: CFS-C 3–4, frail: CFS-C 5–7

^b^ Robust: FI-CGA ≤ 0.08, prefrail: 0.08 < FI-CGA < 0.25, frail: FI-CGA ≥ 0.25

### Criterion concurrent validity

CFS-C categorisation showed fair agreement (weighted kappa = 0.37) and significant correlation (Kendall’s tau = 0.46) with Fried frailty phenotype. For FI-CGA, strong correlation was achieved between CFS-C and FI-CGA (Kendall’s tau = 0.64). Moderate agreement (weighted kappa = 0.51) and strong correlation (Kendall’s tau = 0.63) were also found between categorisation of CFS-C and FI-CGA. All *p* values were < 0.0001 (Table 3).

### Correlation between CFS-C and other geriatric assessments

CFS-C had significant negative correlation with BI, IADL, 6-m gait speed, hand grip, MMSE and MNA, and significant positive correlation with TUG, CIRS-G, and GDS (Table 4). The correlation between CFS-C and ASM was not significant (Table 4).

**Table 4:** Correlation between CFS-C and other geriatric assessments

	N	Kendall’s tau	*p*-value
BI	221	-0.67	< .0001
IADL	221	-0.68	< .0001
MNA	221	-0.36	< .0001
MMSE	223	-0.39	< .0001
GDS	221	0.38	< .0001
CIRS-G	219	0.42	< .0001
6-m gait speed	211	-0.50	< .0001
TUG	215	0.52	< .0001
Hand grip	217	-0.42	< .0001
ASM	207	-0.09	0.07

*Abbreviations*: *CFS-C* Chinese version of Clinical Frailty Scale, *BI* Barthel Index, *IADL* Instrumental Activities of Daily Living, *MNA* Mini-Nutritional Assessment, *MMSE* Mini-Mental State Examination, *GDS* Geriatric Depression Scale, *CIRS-G* Cumulative Illness Rating Scale for Geriatrics, *TUG* Timed-up and go, *ASM* Appendicular skeletal muscle mass

Kendall’s tau was used to assess correlation between CFS-C and other geriatric assessments. *p* < 0.05 was set as statistically significance

### Sensitivity analyses

Different prefrail and frail cutoff points (CFS-C = 4–6) were used for frailty categorisation. Criterion concurrent validity between CFS-C categorisation and frailty phenotype was in fair agreement and significant correlation range (weighted kappa = 0.21–0.29, Kendall’s tau = 0.42–0.44). For validity between categorisation of CFS-C and FI-CGA, the results were in range of fair to moderate agreement and strong correlation (weighted kappa = 0.32–0.43, Kendall’s tau = 0.57–0.63). All *p* values were < 0.0001 [see Additional file 2].

## Discussion

The Chinese version of CFS demonstrated a satisfactory validity and inter-rater reliability for frailty evaluation in Chinese older adults. It was also significantly correlated with various domains of CGA, including function, comorbidity, physical performance, nutrition, cognition and depression, indicating CFS to be a global and synthesis assessment of frailty. Development of a valid CFS-C promotes cross-cultural research of frailty in different populations.

In the 7-point CFS study reported by Rockwood and colleagues, CFS showed high correlation with FI [15]. Meanwhile, in our previous 7-point CFS Chinese version validation study, this tool showed significant agreement and correlation with frailty phenotype [27]. In our current study, we compared the 9-point CFS-C with both FI and frailty phenotype, showing that while there was a significant correlation with both, the correlation was higher with FI. The differential extent of agreement and correlation may reflect distinct concepts between frailty phenotype and FI [14]. Frailty phenotype defines frailty as specific components which constitute energetics and reserve dysregulation [2]. In contrast, FI emphasizes less on specific physical factors and focuses more on accumulation of health deficits [44].

CFS was evaluated as a summarized score after a comprehensive geriatric assessment. The content of CFS gathered information from several domains, including functional disability, comorbidity, cognition, physical activities and self-rated health. Our results of significant correlation between CFS-C and various geriatric conditions were in accordance with those elements and previous studies [15, 17, 27, 45]. Among them, BI and IADL showed the strongest correlation with CFS-C, as function disabilities are important decision points in the CFS classification tree [46]. In addition, we found CFS-C showed significant correlation with grip strength and walking speed, both of which are components of sarcopenia and frailty phenotype. However, no significant correlation was found with muscle mass, the core diagnostic component of sarcopenia. This finding was consistent with a previous study that low muscle mass was more prevalent in patients with sarcopenia than with frailty [47].

Previous studies used CFS of 3 to 6 as the frailty cut-off point with a scale of 5 being the most widely used [17]. In addition, few studies explored the cut-off point for prefrail categorisation for CFS. In sensitivity analyses, we used different cut-off points for prefrail and frailty categorisations. Higher agreement and correlation were achieved when using CFS-C of 3–4 as prefrail categorisation and CFS-C >  = 5 as frailty cut-off point in our study.

CFS was recently updated to version 2.0 [48]. Our team subsequently translated CFS 2.0 into Chinese by the same process as had been used for CFS 1.2 [see Additional file 3]. In agreement with previous study, we found minor differences between the two versions which, in the end, did not bring significant change in grading frailty [49]. Therefore, our results may still apply to CFS 2.0.

Our study has several strengths. First, we followed the standard translation model to develop CFS-C in order to minimize bias. Second, two main frailty assessment instruments, frailty phenotype and FI, were set as references for criterion concurrent validity. Therefore, our design was more appropriate than using only one tool or other surrogates of frailty as reference to measure validity. Third, we used BabyBot vital data recording system to provide user-friendly service and include detailed assessments to compute FI-CGA.

Our study has some limitations. First, uneven distribution of CFS with low percentage of CFS category 1 and lack of category 8–9 limits the external validity, which may partially be a result of our enrollment criteria. However, our results correspond to the characteristics of patients from geriatric clinics, being more complex and having more geriatric syndromes than general older populations. In Taiwan, elders who are categorised as very severely frail or terminally ill (CFS = 8–9) may receive home care, hospice or more frequent inpatient services. Second, the single-center and clinic-based design also limit the generalizability of our results. Validation of CFS-C in other settings will be needed to enhance external validity. Further analysis of our longitudinal cohort to explore predictive validity of CFS-C with different outcomes such as falls, hospitalization and mortality is also warranted.

## Conclusions

In conclusion, the Chinese version of CFS is a valid tool for frailty assessment in Chinese older adults. Development of CFS-C enhanced consistency and accuracy of frailty assessment both in research and clinical practice.

## Supplementary Information

**Additional file 1.** Selection of study participants.

**Additional file 2.** Sensitivity analyses of different CFS-C categorisations.

**Additional file 3.** The Clinical Frailty Scale 2.0 in English (left) and the Chinese translation (right).

## Data Availability

The datasets used and/or analysed during the current study are available from the corresponding author on reasonable request.

## Abbreviations

CFS: Clinical Frailty Scale
CFS-C: Chinese version of CFS
FI-CGA: Frailty Index based on Comprehensive Geriatric Assessment
FI: Frailty Index
CSHA: Canadian Study of Health and Aging
COVID-19: Coronavirus disease 2019
CGA: Comprehensive geriatric assessment
TUG: Timed-up and go
MMSE: Mini-Mental State Examination
GDS-15: Geriatric Depression Scale-15
MNA: Mini-Nutritional Assessment
BI: Barthel Index
IADL: Instrumental Activities of Daily Living
CIRS-G: Cumulative Illness Rating Scale for Geriatrics
ASM: Appendicular skeletal muscle mass
BMI: Body Mass Index
