# Supplementary material for: Validation of clinical frailty scale in Chinese translation
Source: BMC Geriatr. 2022 Jul 20;22:604. doi: 10.1186/s12877-022-03287-x (PMC9298166; doi:10.1186/s12877-022-03287-x)
Supplement: Supplementary file 1 — Additional file 1. Selection of study participants. [file 12877_2022_3287_MOESM1_ESM.docx]

**Additional file 1.** Selection of study participants

**
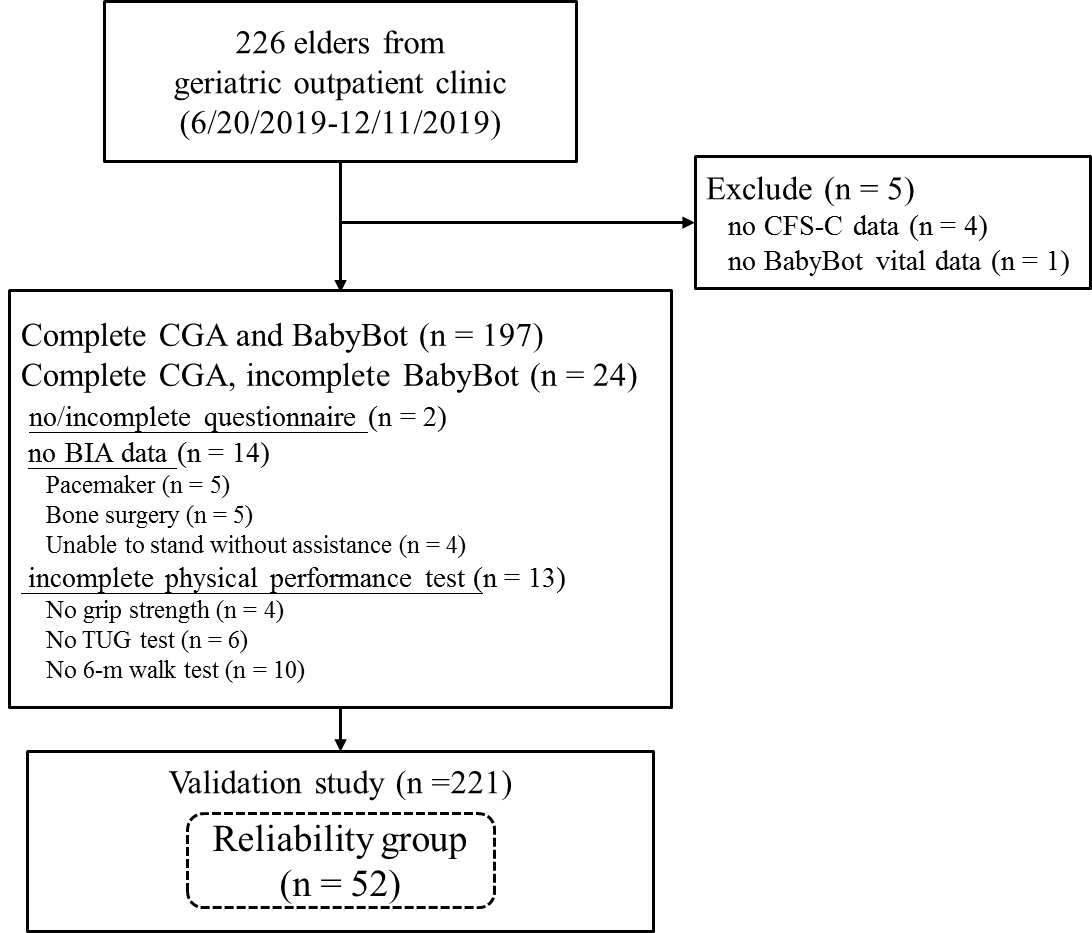
**

**Abbreviations**: CFS-C, Chinese version of Clinical Frailty Scale; CGA, comprehensive geriatric assessment; BIA, bioelectrical impedance analysis; TUG, timed-up and go; 6-m, 6-meter.
