# Supplementary material for: Validation of clinical frailty scale in Chinese translation
Source: BMC Geriatr. 2022 Jul 20;22:604. doi: 10.1186/s12877-022-03287-x (PMC9298166; doi:10.1186/s12877-022-03287-x)
Supplement: Supplementary file 2 — Additional file 2. Sensitivity analyses of different CFS-C categorisations. [file 12877_2022_3287_MOESM2_ESM.docx]

**Additional file 2**. Sensitivity analyses of different CFS-C categorisations

| Tests | N | Weighted kappa | *p*-value | Kendal’s tau | *p*-value |
| --- | --- | --- | --- | --- | --- |
| Criterion concurrent validity |  |  |  |  |  |
| CFS-C categorisation^a^ vs. Fried frailty phenotype | 213 | 0.28 | <.0001 | 0.42 | <.0001 |
| CFS-C categorisation^b^ vs. Fried frailty phenotype | 213 | 0.21 | <.0001 | 0.42 | <.0001 |
| CFS-C categorisation^c^ vs. Fried frailty phenotype | 213 | 0.29 | <.0001 | 0.44 | <.0001 |
| CFS-C categorisation^a^ vs. FI-CGA categorisation^d^ | 214 | 0.38 | <.0001 | 0.57 | <.0001 |
| CFS-C categorisation^b^ vs. FI-CGA categorisation^d^ | 214 | 0.32 | <.0001 | 0.60 | <.0001 |
| CFS-C categorisation^c^ vs. FI-CGA categorisation^d^ | 214 | 0.43 | <.0001 | 0.63 | <.0001 |

^a^Robust: CFS-C 1-2, prefrail: CFS-C 3-5, frail: CFS-C 6-7.

^b^Robust: CFS-C 1-3, prefrail: CFS-C 4-5, frail: CFS-C 6-7.

^c^Robust: CFS-C 1-3, prefrail: CFS-C 4, frail: CFS-C 5-7.

^d^Robust: FI-CGA ≤ 0.08, prefrail: 0.08 < FI-CGA < 0.25, frail: FI-CGA ≥ 0.25.

**Abbreviations**: CFS-C, Chinese version of Clinical Frailty Scale; FI-CGA, Frailty Index based on a Comprehensive Geriatric Assessment.
