# Supplementary figures and images for: Validation of clinical frailty scale in Chinese translation
Source: BMC Geriatr. 2022 Jul 20;22:604. doi: 10.1186/s12877-022-03287-x (PMC9298166; doi:10.1186/s12877-022-03287-x)

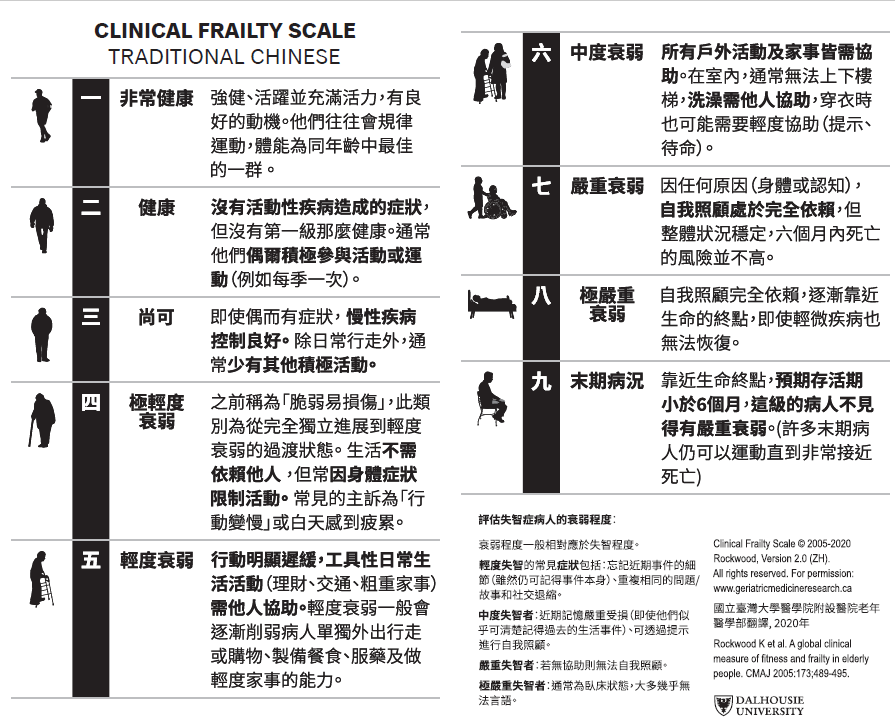
**
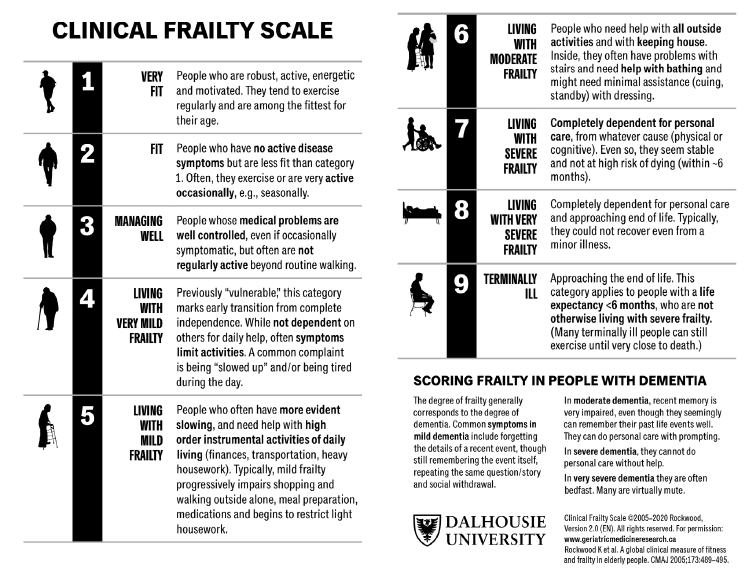
Additional file 3.** TheClinical Frailty Scale 2.0 in English (left) and the Chinese translation (right)

Supplement: Supplementary file 3 — Additional file 3. The Clinical Frailty Scale 2.0 in English (left) and the Chinese translation (right). [file 12877_2022_3287_MOESM3_ESM.docx]
